# Establishment of rapid and non-invasive protocols to identify B-carrying individuals of *Psalidodon paranae*

**DOI:** 10.1590/1678-4685-GMB-2020-0003

**Published:** 2021-03-26

**Authors:** Caio Augusto Gomes Goes, Duílio Mazzoni Zerbinato de Andrade Silva, Ricardo Utsunomia, George Shigueki Yasui, Roberto Ferreira Artoni, Fausto Foresti, Fábio Porto-Foresti

**Affiliations:** 1 Universidade Estadual Paulista “Júlio de Mesquita Filho” (UNESP), Faculdade de Ciências, Bauru, SP, Brazil; 2 Universidade Estadual Paulista “Júlio de Mesquita Filho” (UNESP), Instituto de Biociências, Botucatu, SP, Brazil; 3 Universidade Federal Rural do Rio de Janeiro, Instituto de Ciências Biológicas e da Saúde, ICBS, Seropédica, RJ, Brazil; 4 Centro nacional de Pesquisa e Conservação da Biota Aquática Continental (CEPTA-ICMBIO), Pirassununga, SP, Brazil; 5 Universidade Estadual de Ponta Grossa, Setor de Ciências Biológicas e da Saúde, Ponta Grossa, PR, Brazil

**Keywords:** Psalidodon, Neotropical fishes, satellite DNA, qPCR, B chromosomes

## Abstract

Supernumerary, or B, chromosomes are present in several eukaryotes, including characid fish of the genus *Psalidodon*. Notably, *Psalidodon paranae* carries the most studied B chromosome variant, a macro-B chromosome. The origin of this element was determined to be an isochromosome; however, data regarding its inheritance remain unavailable due to methodological barriers such as the lack of an efficient, non-invasive, and rapid protocol for identifying B-carrying individuals that would enable the design of efficient crossing experiments. Thus, in this study, we primarily aimed was to develop two non-invasive and fast (approximately 2 h) methods to identify the presence of B chromosomes in live specimens of *P. paranae* based on satellite DNA (satDNA) sequences known to be present in this element. The methods include fluorescence *in situ* hybridization in interphase nuclei and relative gene quantification of satDNAs using quantitative polymerase chain reaction. Our results reveal the efficiency of quick-fluorescence in situ hybridization and quantitative polymerase chain reaction for identifying B-carrying individuals using the proposed satDNA sequences and open up new possibilities to study B chromosomes.

Supernumerary or B chromosomes are dispensable chromosomes additional to the standard A complement (Jones and Rees, 1982; Camacho *et al.*, 2000). These elements can be found in diverse species (including plants, animals, and fungi) (Camacho *et al.*, 2000; Burt and Trivers, 2006; Ahmad and Martins, 2019), representing approximately 15% of eukaryotes. In general, B chromosomes do not follow a Mendelian segregation pattern and do not always occur in pairs, which can facilitate inheritance values higher than 0.5, resulting in transmission advantages, or the so-called “drive” process (Houben, 2017).

In fish, B chromosomes have been reported in approximately 113 species, of which 38 are found in the Neotropical region, such as characid fish of the genus *Psalidodon* (D’Ambrosio *et al.*, 2017). Remarkably, one can observe a wide variety of B chromosomes in *Psalidodon,* with primary differences in their morphology, ranging from large metacentric chromosomes similar in size to the first pair of chromosomes in *P. scabripinnis* and *P. paranae* (Salvador and Moreira-Filho, 1992; Silva *et al.*, 2014) to microchromosomes, in *P. bockmanni* (Daniel *et al.*, 2012).

During the preceding decades, the large metacentric B chromosome variant, or the macro-B chromosome, observed in seven species of *Psalidodon*, was analyzed using distinct approaches (Maistro *et al.*, 1992; Salvador and Moreira-Filho, 1992; Moreira-Filho *et al.*, 2001; Torres-Mariano and Morelli, 2008; Silva *et al.*, 2016). These species were originally part of the *Astyanax* genus, but a recent revision led to their inclusion in the *Psalidodon* genus (Terán *et al.*, 2020). Fluorescence in situ hybridization (FISH) patterns of repetitive DNAs revealed that this B chromosome is an isochromosome, of intraspecific origin, of the A complement in *P. paranae* and *P. scabripinnis* (Mestriner *et al.*, 2000; Silva *et al.*, 2014). In addition, chromosome painting data revealed a common origin of this macro-B chromosome for several species, with subsequent independent diversification processes (Vicari *et al.*, 2011; Silva *et al.*, 2016; 2017). *P. paranae* has the highest population frequency of B chromosomes in the genus, with approximately 40% of individuals carrying them (Porto-Foresti *et al.*, 1997). Furthermore, the accumulated data from next-generation sequencing of B-carrying (1B) and non-B-carrying (0B) individuals, obtained through comparative analysis of these two genomes (Silva *et al.*, 2017), show *P. paranae* to be a model for studying B chromosomes of the genus *Psalidodon*. Although the origin and evolution of this genomic element were assessed several times using multiple approaches, its inheritance patterns have never been studied, even though such an analysis could assist in understanding the life cycle of this chromosome. Therefore, the development of non-invasive protocols for genotyping B-carrying individuals of *P. paranae* would allow efficient designing of crossing experiments that could uncover myriad mating possibilities for this species.

There is a fundamental need to develop non-invasive methods for identification of fish species that are aimed at preserving individuals without disturbing their integrity. These methods can be applied in many ways, such as measuring the size of internal organs (Näslund, 2014), characterizing immune responses (Matsuura *et al.*, 2017), and identifying the sex of individuals (Utsunomia *et al.*, 2017; 2019). Thus, the standardization of non-invasive protocols to identify 1B and 0B individuals of *P. paranae* will allow a glimpse into the inheritance patterns of B chromosomes in fish. In this context, the existence of clustered satellite DNA (satDNA) sequences on B chromosomes of this species provides an opportunity to develop a FISH-based method that uses these repetitive sequences.

In this study, 10 individuals from *P. paranae* (six females and four males) were collected from Cascatinha Stream, Botucatu, São Paulo, Brazil (22°53′30″S 48°28′36″W) with authorization of the relevant organizations (MMA / IBAMA / ICMBio / SISBIO - 18884-1, registered with IBAMA No. 2567470). The procedures, maintenance, and analysis of all fish were performed in compliance with the standards of the Brazilian College of Animal Experimentation. The individuals were deposited in the collection of the Laboratório de Genética de Peixes at UNESP, Bauru, São Paulo, Brazil under voucher LGP 10120 - 10130. Small fragments of fins from each individual were cut and preserved in 96% alcohol and Carnoy’s solution (methanol-acetic acid, 3:1) for laboratory analysis. Then, 15 µL of cell suspension was dispensed on the slides and dried at 37 ºC for 30 min. The individuals were then sacrificed by treatment with an overdose of anesthetic in 1% benzocaine and were later dissected. Next, mitotic chromosomes were obtained from all the sample specimens according to the protocol established by Foresti *et al.* (1981) to visually confirm the presence of B chromosomes.

The Apasat06-86 satDNA described by Silva *et al.* (2017) was selected to detect the presence of B chromosomes because the hybridization patterns of the satDNA showed the presence of a large pericentromeric block in the B chromosomes of this species (Silva *et al.*, 2017). FISH experiments in interphase cells were performed according to the protocol established by Utsunomia *et al.* (2019), with 30 min probe hybridization. The probes were generated as described by Silva *et al.* (2017), and probe detection was performed using anti-digoxigenin-rhodamine (Roche). Chromosomes and interphase nuclei were counterstained using 4’, 6-diamidino-2-phenylindole (Vector Laboratories). The entire process takes approximately 2 h. To verify the efficiency of our methods, approximately 100 interphase nuclei were analyzed.

In addition to the quick-FISH method, a quantitative polymerase chain reaction (qPCR)-based method was performed in parallel, to independently confirm the presence of B chromosomes in the *P. paranae* individuals. The copy numbers of the three satDNA sequences present on B chromosomes of *P. paranae* were determined using relative quantification (RQ). ApaSat06-86 was used for the quick-FISH experiment, whereas ApaSat20-18 and ApaSat44-21 were tested for their presence exclusively on the B chromosomes in this species using FISH (Silva *et al.*, 2017).

Total DNA was extracted from fin fragments of the individuals using the Wizard Genomics Kit (Promega). qPCR was performed using the StepOne Real-Time PCR System (Life Technologies, Carlsbad, CA, USA). We used the 2^-∆Ct^ method (Bel *et al.*, 2011) for RQ, using the single-copy gene *hypoxanthine phosphoribosyltransferase* (*Hprt*) as reference. The primers described by Silva *et al.* (2017) for satDNAs and Utsunomia *et al.* (2019) for *Hprt* were used. DNA from three samples each from the 0B and 1B groups were analyzed simultaneously using three independent replicates for both the target and reference genes. The reactions were performed in a final volume of 10 µL with 3 ng of genomic DNA, 5 μL of Power SYBR™ Green PCR Master Mix (Applied Biosystems) and 1 μL of each 5 µM primer. The cycle conditions were as follows: 95 °C for 10 min and 45 cycles of 95 °C for 15 s and 60 °C for 1 min. The specificity of the PCR products was confirmed using dissociation curve analysis. No samples were discarded as the Ct values showed a consistent pattern. The RQ value is represented as the mean ± standard error of the mean. Statistical analysis was performed using the Shapiro-Wilk test to ascertain whether the variables fit a normal distribution, followed by the Student’s *t*-test. A summary of the methodology is shown in Figure 1.

**Figure 1 -:**
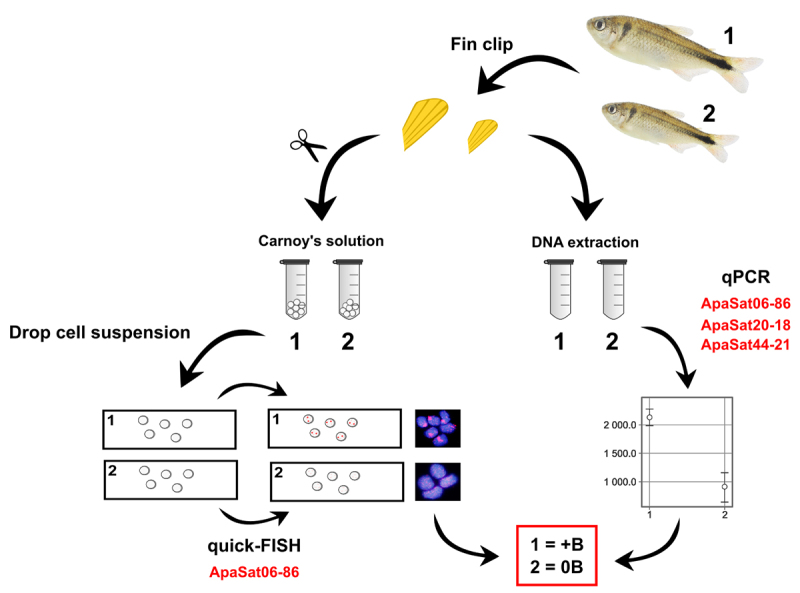
Schematic summary of the practical steps proposed in this study to identify B-carrying individuals of *Psalidodon paranae.*

FISH in metaphase cells of *P. paranae* revealed the discrete presence of the Apasat06-86 satDNA sequence in the centromeric and telomeric regions of some chromosomes in the A set. In 1B individuals, FISH revealed a large accumulation of this satDNA sequence in the centromeric region of B chromosomes (Figure 2a-c), corroborating the results obtained by Silva *et al.* (2017). FISH in interphase cells showed several small signals distributed throughout the cell nuclei in 0B individuals, corresponding to the sites observed in the chromosome of the standard set (Figure 2d-f). However, 1B individuals also showed a large and conspicuous mark in their nuclei (Figure 2g-i). Thus, the Apasat06-86 probe was 100% efficient in identifying 1B individuals and eliminated the necessity for a control probe. Our microscopic analysis of 85% of the cells from each individual presented the expected signal patterns. However, it should be noted that this satDNA sequence is not suitable for qPCR experiments because of its accumulation in chromosomes other than B chromosomes (Figure 3).

**Figure 2 -:**
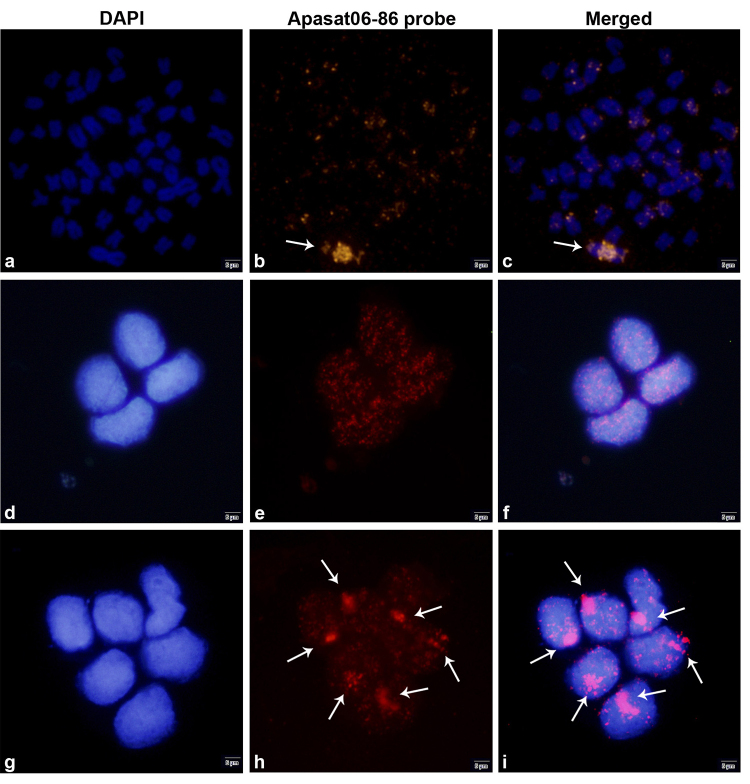
Signal patterns of signals obtained for the Apasat06-86 satellite after fluorescent in situ hybridization (FISH) in *Psalidodon paranae*. This sequence displays a conspicuous block corresponding to the B chromosomes of this species shown using arrows, while small signals scattered throughout normal chromosomes can also be seen (b, e, h). It also depicts FISH in the interphase nuclei of non-B-carrying (0B), in which only small-scattered signals are observed (e, f). The same technique performed on B-carrying (1B) individuals illustrates the presence of scattered signals in addition to a large block corresponding to B chromosome (h, i).

**Figure 3 -:**
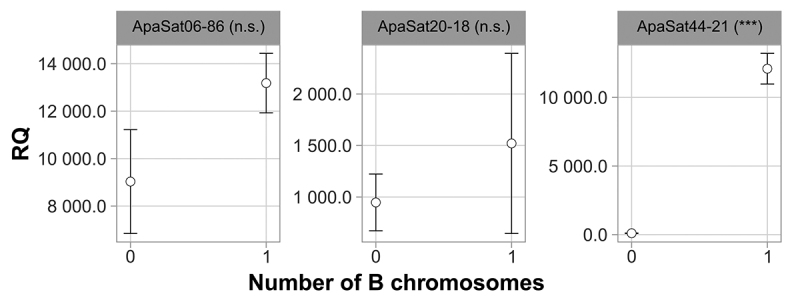
Relative quantification (RQ) of three satellite DNAs from non-B-carrying (0B) and B-carrying (1B) individuals of *Psalidodon paranae*. n.s.: p > 0.05. ***: p < 0.001.

Conventional PCR was performed at intervals of 5, 10, and 15 cycles to verify the amplification of the Apasat06-86 sequence. Except for 5-cycle reactions, samples from both 0B and 1B individuals demonstrated the expected banding patterns (Figure 4), confirming that a PCR-based method is not suitable for genotype individuals of *P. paranae*. To overcome this limitation, ApaSat44-21 was tested and confirmed to be an efficient marker, showing a significant difference in genomic abundance when comparing 0B and 1B individuals (Figure 3). FISH mapping of this satDNA confirmed its clustering only in the B chromosomes of *P. paranae,* and not in the genomes of *P. fasciatus* or *P. bockmanni* (Silva *et al.*, 2017). qPCR using the other two proposed satellites did not show any significant differences between 0B and 1B individuals, probably due to their presence in A chromosomes (Figure 3).

**Figure 4 -:**
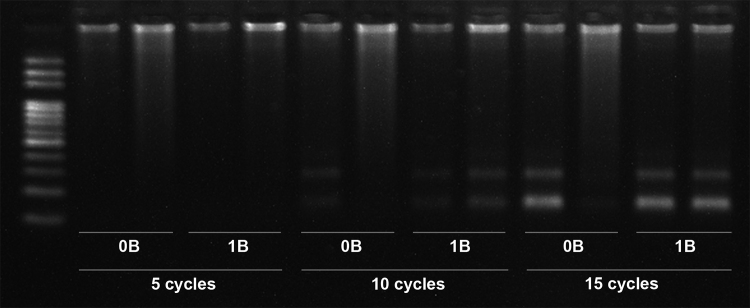
Agarose gel showing Apasat06-86 sequence amplification. No significant differences were observed in band patterns of non-B-carrying (0B) and B-carrying (1B) individuals after 15 cycles of conventional polymerase chain reaction.

The complete satellitome of *P. paranae* was described by Silva *et al.* (2017), and FISH mapping demonstrated the presence of 15 satDNA sequences in the B chromosomes of this species. In addition, several of these satDNAs are shared by the B chromosome of *P. bockmanni* and *P. fasciatus* (Silva *et al.*, 2017), despite the fact that next-generation sequencing data are not available for these two species. In this case, any of the satDNAs shared between the B chromosomes of *P. paranae* and the variants present in *P. fasciatus* and *P. bockmanni* have the potential to function as markers of B chromosomes. It is possible to replicate the method, which used the Apasat06-86 satDNA, proposed in this study to identify B chromosomes in *P. paranae* utilizing the Apasat20-18 satDNA, which demonstrates the pattern of distribution in *P. fasciatus* and *P. bockmanni* (Silva *et al.*, 2017). Despite the potential of this method, B chromosomes are present at a low frequency in *P. fasciatus* and *P. bockmanni*, contrary to what was observed in *P. paranae* (Porto-Foresti *et al.*, 1997).

Our data demonstrate the practical use of satDNAs as potential biotechnological tools for identifying *P. paranae* individuals with supernumerary, or B, chromosomes. Similar applications have already been demonstrated for *Megaleporinus macrocephalus*, in which there is an accumulation of satDNA families on the W sex chromosome (Utsunomia *et al.*, 2019). Here, the non-invasive identification of *P. paranae* individuals with B chromosomes could allow the use of these fish for breeding, with the aim of describing the inheritance patterns of supernumerary elements as well as promoting full population studies for detecting the distribution of these elements in natural individuals, including juveniles.

*P. paranae* is an important species for studying B chromosomes in the genus *Psalidodon* because of the accumulated knowledge regarding its morphology, population distribution, molecular structure, and genetic content (Maistro *et al.*, 1992; Porto-Foresti *et al.*, 1997; Silva *et al.*, 2014, 2016, 2017). Thus, the use of satDNA sequences to develop faster, more effective, and cheaper identification methods that do not require animal sacrifice is extremely important. This work presents two rapid and effective methods for identifying *P. paranae* individuals carrying B chromosomes, and the findings may open new possibilities for the study of the evolutionary dynamics of B chromosomes in *Psalidodon*.
